# Food Hypersensitivity: Diagnosing and Managing Food Allergies and Intolerances

**DOI:** 10.1155/2012/576017

**Published:** 2012-11-11

**Authors:** Carina Venter

**Affiliations:** David Hide Asthma and Allergy Research Centre, Isle of Wight and School of Health Sciences and Social Work, University of Portsmouth, Portsmouth PO1 2FR, UK

The last two years have seen the publication of 3 major guidelines on the diagnosis and management of food hypersensitivity (FHS) [1–3]. These guidelines have highlighted important issues in dealing with those with FHS, primarily food allergies. Dealing with adults and children with food allergies requires the healthcare professional to have an understanding of the immune mechanisms involved in the development of allergy versus tolerance, diagnostic methods, managing the patient, and the development of new treatment modalities. In this issue, we have continued to discuss topics such as the role of the epithelial barrier in the development of FHS, the importance of patient education and support groups, the effect of food production on the allergenicity of foods, and the induction of oral tolerance. This special issue is concluded with a tutorial from expert allergy dietitians on the diagnosis and management of FHS in children.

Food hypersensitivity includes a wide spectrum of symptoms and mechanisms. Many researchers and clinicians have discussed the possible role of the gut (barrier) and the mucosal immune system in the development of food allergies. Factors that have been addressed in particular include weaning and the weaning diet [4], age of solid food introduction and breast feeding [5], gut bacteria [6], and gastrointestinal infections [7]. What is clear is that a better understanding of the immunological mechanisms involved in sensitisation and tolerance will enhance our efforts of allergy prevention and discovering novel treatments. L. C.-H. Yu discusses in the review paper “*Intestinal epithelial barrier dysfunction in food hypersensitivity*” the epithelial barrier dysfunction in sensitized intestines with particular reference to the enhanced transcytotic rates of allergens and the mechanisms involved in development of sensitisation. They discuss that recent studies demonstrate that food allergens manage to be transported across the epithelium and avoid lysosomal degradation by binding to cell surface IgE and the low-affinity receptor CD23/Fc*ε*RII—leading to investigation of anti-IgE and anti-CD23 antibodies in the prevention and management of allergic diseases.

A clear diagnosis plays a very important role in the management of food hypersensitivities. The paper “*Late type of bronchial response to milk ingestion challenge: a comparison of open and double-blind challenge*” by Z. Pelikan addresses two important issues often discussed by allergists and other health care professionals [8]. (1) Can cow's milk cause a bronchial response in patients with diagnosed asthma and (2) can open food challenges be used for the diagnosis of bronchial responses in asthmatics? In this study the authors showed that cow's milk allergy can lead to late-phase bronchial complaints (2 hours after ingestion) in patients with diagnosed asthma either as the sole allergen causing the problem or in addition to other aeroallergens. In addition, they showed that the open food ingestion challenge (OFICH) combined with monitoring of the objective lung function parameters can be considered as a definite confirmation of the suspected role of food allergy and involvement of a certain food (e.g., cow's milk) in bronchial complaints of patients suffering from bronchial asthma due to various inhalants, making the use of DBPCFC superfluous.

It is well known that food hypersensitivity leads to reduced quality of life of both the parent and the child suffering from this [9–11]. The World Allergy Organisation Guidelines [2], the US Food Allergy Guidelines [1], and the UK NICE Guidelines on Food Allergy [3] stated the importance of patient support groups for sufferers of FHS. These support groups are, however, often set up and run by allied health professionals. It is therefore very enlightening to read in the paper “*A pediatric food allergy support group can improve parent and physician communication: results of a parent survey*” by A. Sharma et al. that a food allergy support group decreased anxiety about food allergies for 77.7% of families. Almost 65% of parents indicated that an allergist attending the support group increased their communication with their doctor. The most important reason for joining the support group was to increase their knowledge on food allergies, followed by meeting others with food allergies, reduce their anxiety, recipe ideas, and improve their quality of life.

Patient education on food labelling is another topic of importance highlighted by the food allergy guidelines, with many questions surrounding the issue of allergen content of processed food [12]. P. A. Alvarez and J. I. Boye discussed in their paper “*Food production and processing considerations of allergenic food ingredients: a review*” the importance of epidemiological data, indicating the most allergenic foods in a population. Despite tests available to detect allergens in food and a constant improvement in this science, is allergen risk management right from the primary ingredients is the most important factor in reducing the risk for allergic consumers. This also includes the practice of minimising ingredient and simplifying sourcing of ingredients.

This special issue is concluded with a tutorial by allergy dietitians and guest editors, Drs. Laitinen, Venter, and Vlieg-Boerstra. The tutorial covers a general overview of FHS, addressing in particular nomenclature of food hypersensitivity, prevalence, recently published guidelines, the principles of taking an allergy focussed diet history, and the principles of the nutritional management of FHS.

*Carina Venter* *Carina Venter*
